# Changes in the Physicochemical Properties and Microbial Communities of Air-Fried Hairtail Fillets during Storage

**DOI:** 10.3390/foods13050786

**Published:** 2024-03-03

**Authors:** Yixuan Ding, Yueqin Liao, Jiangyue Xia, Disha Xu, Menghua Li, Hongli Yang, Huimin Lin, Soottawat Benjakul, Bin Zhang

**Affiliations:** 1 Zhejiang Provincial Key Laboratory of Health Risk Factors for Seafood, College of Food and Pharmacy, Zhejiang Ocean University, Zhoushan 316022, China; dyxdhhy@163.com (Y.D.); liaoyueqin0225@163.com (Y.L.); xiajiangyue9@163.com (J.X.); 18868008010@163.com (D.X.); lmh15005726451@163.com (M.L.); yanghongli79@163.com (H.Y.); zhangbin@zjou.edu.cn (B.Z.); 2 International Center of Excellence in Seafood Science and Innovation, Faculty of Agro-Industry, Prince of Songkla University, Hat Yai, Songkhla 90112, Thailand; soottawat.b@psu.ac.th; 3 Pisa Marine Graduate School, Zhejiang Ocean University, Zhoushan 316022, China

**Keywords:** hairtail fillets, air frying, microbial diversity, physicochemical properties

## Abstract

This study assessed the physicochemical properties of air-fried hairtail fillets (190 °C, 24 min) under different storage temperatures (4, 25, and 35 °C). The findings revealed a gradual decline in sensory scores across all samples during storage, accompanied by a corresponding decrease in thiobarbituric acid reactive substances (TBARS) and total viable count over time. Lower storage temperatures exhibited an effective capacity to delay lipid oxidation and microbiological growth in air-fried hairtail fillets. Subsequently, alterations in the microbiota composition of air-fried hairtail fillets during cold storage were examined. Throughout the storage duration, *Achromobacter*, *Escherichia-Shigella*, and *Pseudomonas* emerged as the three dominant genera in the air-fried hairtail samples. Additionally, Pearson correlation analysis demonstrated that among the most prevalent microbial genera in air-fried hairtail samples, *Achromobacter* and *Psychrobacter* exhibited positive correlations with the L* value, a* value, and sensory scores. Conversely, they displayed negative correlations with pH, b* value, and TBARS. Notably, air-fried samples stored at 4 °C exhibited prolonged freshness compared with those stored at 25 °C and 35 °C, suggesting that 4 °C is an optimal storage temperature. This study offers valuable insights into alterations in the physicochemical properties and microbial distribution in air-fried hairtail fillets during storage, facilitating the improvement of meat quality by adjusting microbial communities in air-fried hairtail fillets.

## 1. Introduction

The hairtail (*Trichiurus lepturus*), a highly prized marine fish species, is widely distributed across the eastern Pacific Ocean and along the coastlines of various regions in China [1]. Among China’s four major commercial fish species, the hairtail is notably rich in vitamins, minerals, polyunsaturated fatty acids, proteins, and essential amino acids, such as eicosatetraenoic acid and docosahexaenoic acid [2,3]. Consequently, several hairtail products, including air-dried hairtail [4], vinasse hairtail fillets [5], and deep-fried hairtail fillets [6], have been developed. Deep-fried hairtail fillets, a renowned traditional Chinese fried product, are highly favored by Chinese consumers due to their distinct taste, flavor, and crisp texture. However, the excessive oil content in deep-fried products dissuades some consumers [7]. Numerous studies have linked health concerns such as obesity, coronary heart disease, and hypertension to excessive oil consumption [8]. Therefore, studies [9,10,11] aimed at reducing the oil content in fried products while maintaining product quality (texture, flavor, taste, and appearance) are imperative, especially considering the formation of carcinogenic compounds, such as acrylamide and PAHs in fried foods, which pose significant health risks.

To address these concerns and explore healthier alternatives in food preparation methods, recent research has focused on air frying as an alternative to deep frying. Air frying is an innovative cooking technique that uses hot air circulation to cook food evenly and ensure contact between food components [12]. This method, using superheated air circulation instead of hot oil, cooks food faster than conventional oven baking [13]. Moreover, air-fried food exhibits a crisp exterior and a moist interior, offering sensory qualities comparable to those achieved through deep-frying methods [14]. A previous study by Santos et al. [15] demonstrated that air-fried potatoes yield significantly fewer trans fatty acids than those deep-fried. However, existing research has predominantly focused on the biochemical alterations in air-fried products. For example, Ding et al. [6] conducted a comparative analysis of the physicochemical and volatile flavor attributes of deep-fried and air-fried hairtail fillets. Similarly, Qin et al. [16] explored the formation of advanced glycation end products in fish cakes during the air-frying process. However, to our knowledge, minimal studies have examined the intricate microbial community and physicochemical attributes of air-fried hairtail fillets during storage. Furthermore, there has been a dearth of research examining the relationship between physicochemical qualities and microbial composition in this context.

Traditional methods for detecting microorganisms in meat and meat products rely on widely used cultivation-based approaches, which analyze less than 1% of the total bacterial population [17]. Alternatively, culture-independent techniques offer more sophistication and reliability when evaluating microbial communities. For instance, PCR-denaturing gradient gel electrophoresis (PCR-DGGE) is a commonly-used molecular technique for studying microbiota alterations during the storage of meat and meat products [18].

However, PCR-DGGE has limitations, including restricted resolution and significant background interference [19]. Presently, the study of food fermentation or spoiling extensively employs 16S rRNA amplicon high-throughput sequencing (HTS) technology [20]. HTS allows for quick and accurate identification of the majority of microorganisms detected in food samples by analyzing thousands of sequences. Understanding how microbiota changes over time in food ecology is crucial [21]. Therefore, utilizing HTS to evaluate the microbial diversity of air-fried hairtail fillets offers a more comprehensive and accurate assessment. The microbial community structure during storage can influence the physicochemical qualities, thereby affecting the final product’s quality and safety.

Consequently, this study examines the effect of storage temperature on quality indicators (pH, color, thiobarbituric acid reactive substance (TBARS), sensory evaluation, and total viable count (TVC)) in air-fried hairtail fillets. Additionally, it delves into the microbial composition of these samples, aiming to contribute to the theoretical basis for improving product safety and extending the shelf life of air-fried hairtail fillets during storage.

## 2. Materials and Methods

### 2.1. Materials

Hairtail samples (450 ± 50 g, 63.0 ± 2.0 cm) were procured from a local fishery market in Zhoushan (Zhejiang, China) and promptly transported to the laboratory on flake ice within 25–30 min. Trichloroacetic acid was obtained from the Shanghai Aladdin Biochemical Technology Co., Ltd. (Shanghai, China), while 2-thiobarbituric acid was obtained from the Guangzhou Chemical Reagent Factory (Tianjin, China).

### 2.2. Sample Preparation and Storage

The hairtail was stored at 4 °C. After the removal of the head, fins, viscera, and tail (without bones), the samples were washed with distilled water and cut into fillets (6.0 cm). The air fryer (Airfryer Philips HD9741 Xinan Technology (Shenzhen) Co., Ltd., Shenzhen, China) was preheated for 5 min at 190 °C, after which the fish fillets were placed in the cooking basket of the air fryer equipment. The air fryer program was set for 190 °C and 24 min. The air-fried fish samples were then allowed to cool at room temperature for 30 min before being vacuum-sealed in a polyethylene bag, using a vacuum packaging machine (DZ-400-2S, Shanghai Xinqi Machinery Group Co., Ltd., Shanghai, China).

Finally, some of the packaged samples were stored in a refrigerator (4 °C) for 12 days. Physicochemical measurements were conducted at 0, 3, 6, 9, and 12 days. Meanwhile, the remaining two packaged samples were stored in an electric thermostatic incubator at 25 and 35 °C for 9 and 4 days, respectively. Measurements of the physicochemical characteristics were performed at 0, 2, 4, 6, and 8 days and 0, 1, 2, 3, and 4 days, respectively. Additionally, one raw and three air-fried fish samples (0, 6, and 12 days), subjected to HTS technology during cold storage, were preserved at −80 °C (MDF-U32V(N), SANYO Electric Co., Ltd., Osaka, Japan).

### 2.3. Determination of Color, pH, and Thiobarbituric Acid Reactive Substance

The color profile of the hairtail muscle was measured using a CS-210 precise colorimeter (Hangzhou CHNSpec Technology Co., Ltd., Hangzhou, China). Calibration of the colorimeter was conducted before each measurement using standard white and black plates.

To measure the pH, 5.0 g of minced sample was mixed with 50 mL of distilled water for 30 min, then filtered, and the pH of the resulting filtrate was determined using an S210 pH meter (Mettler-Toledo (China) Ltd., Shanghai, China).

To assess the TBARS value in air-fried hairtail samples, the procedure followed the National Food Safety Standard Method of China (Determination of malonaldehyde in food; GB, 5009.181, 2016). The results were expressed as milligrams of MDA per kilogram of sample, and all samples underwent triplicate testing.

### 2.4. Determination of Total Viable Count

The TVC of air-fried fish samples was evaluated according to the method previously described by Yang et al. [22], with slight modifications. A 5.0 g portion of minced sample was homogenized for 1 min using a stomacher (Clapping Stomacher NAI-JZQ1, Shanghai Naai Laboratory Instrument Co., Ltd., Shanghai, China) in 45 mL of 0.85% saline solution. The homogenate was serially diluted, and 1 mL of each dilution was spread onto the surface of a plate count agar (Luqiao Co. Ltd., Beijing, China). After incubation at 37 °C for 48–72 h, colony counts were conducted, and results were expressed as logarithm base 10 colony-forming units per gram (log CFU/g).

### 2.5. Sensory Assessment

The sensory analysis of the air-fried fish samples was conducted by 10 experienced panelists, consisting of five women and five men, aged 21–27 years. Following the method of Xie et al. [23], with some modifications, we evaluated overall acceptability based on color, odor, and texture. Scores ranged from 1 to 2 for intense dislike, 3 to 5 for unacceptable quality, 6 to 8 for acceptable quality, and 9 to 10 for good quality. The evaluation was performed in a separate room illuminated with white light, allowing panelists access to drinkable water to refresh their palates during the examination.

### 2.6. Microbial Diversity Analysis

Bacterial genomic DNA was extracted using a Fast DNA SPIN extraction kit (MP Biomedical, Santa Ana, CA, USA), following the manufacturer’s protocol. To ensure the purity and quality of the extracted DNA, both 1% agarose gel electrophoresis and spectrophotometric analysis using a NanoDrop^®^ ND-2000 spectrophotometer (Thermo Scientific Inc., Waltham, MA, USA) were employed.

For amplification of the internal transcribed spacer (ITS) region of prokaryotic (bacterial) DNA, a slightly modified version of primers V3–V4 was employed. This modification involved using primers 338 F (5′-ACTCCTACGGGAGGCAGCAG-3′) and 806 R (5′-GGACTACHVGGGTWTCTAAT-3′) tagged with different barcodes. The PCR amplification process was performed on an ABI GeneAmp^®^ 9700 PCR thermocycler (ABI, Vernon, CA, USA). The PCR mixture comprised 20 μL, including 4 μL of 5× Fast Pfu buffer, 2 μL of 2.5 mM dNTPs, 0.8 μL of each primer (5 μM), 0.4 μL of Fast Pfu polymerase, 0.2 μL of BSA, 10 ng of template DNA, and an appropriate volume of double-distilled water. The resulting PCR amplification products were purified using the AxyPrep DNA Gel Extraction Kit (Axygen Biosciences, Union City, CA, USA), following the manufacturer’s provided protocol. Subsequently, the quantification of the amplicons was conducted using QuantiFluor™-ST (Promega, Madison, WI, USA), as per the manufacturer’s guidelines.

Paired-end sequencing of equimolar purified amplicons was performed using an Illumina MiSeq platform (Illumina, San Diego, CA, USA). This sequencing procedure was performed by Majorbio Bio-Pharm Technology Co. Ltd. (Shanghai, China), following standard protocols. The raw sequence reads were demultiplexed and quality-filtered using Quantitative Insights into Microbial Ecology software (QIIME version 1.7.0) [24]. In summary, during data processing with QIIME 1.7.0, raw sequencing reads were initially sorted based on their matching barcodes, and those matches were considered valid sequences attributed to their respective samples. Subsequently, after chimera identification, the remaining sequences were categorized as effective sequences, ready for further analysis. The effective sequence’s similarity was used by UCLUST [25] to identify it, and those with a 97% similarity were grouped into a single operational taxonomic unit (OTU).

### 2.7. Data Analysis

The experiments in this study were performed in triplicate to ensure the reliability of the results. Statistical analysis was conducted using SPSS 25.0 (SPSS Inc., Chicago, IL, USA) for one-factor ANOVA and correlation analysis. Graphs and data visualization were created using Origin 2021. To identify statistically significant differences, Duncan’s multiple comparison tests were employed, with a significance threshold set at *p* < 0.05. This threshold is commonly accepted to indicate statistical significance in the obtained results.

## 3. Results and Discussion

### 3.1. Physicochemical Characteristics

**Figure 1:**
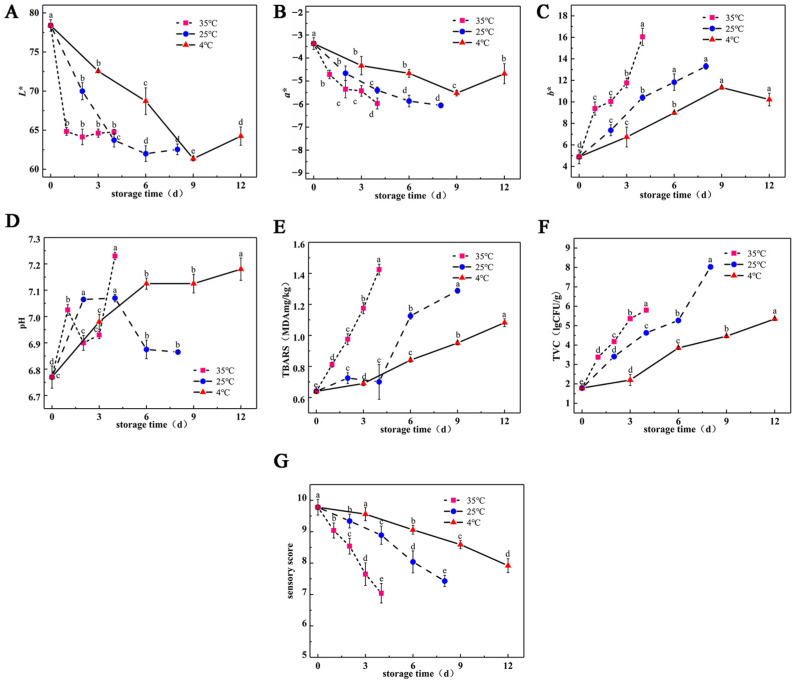
Changes in color (**A**–**C**), pH (**D**), TBARS (**E**), TVC (**F**), and sensory score (**G**) of air-fried hairtail fillets during storage. Different letters within each substrate indicate significant difference (*p* < 0.05).

Figure 1 illustrates the physicochemical characteristics of air-fried hairtail fillet samples. Meat color is a significant factor influencing consumer purchase decisions due to its visual appeal. The color variations of these fillets, stored at different temperatures, are depicted in Figure 1A–C. The initial color profile of freshly air-fried samples provides valuable insights: lightness (L*), measured at 78.40; redness (a*), at −3.37; and yellowness (b*), at 4.89. During the storage period, all samples exhibited a decreasing trend in L* values, indicating a shift from brighter to darker hues, with higher temperatures accelerating this change. This may be attributed to protein and fat denaturation, leading to increased light absorption by air-fried hairtail fillets [26]. The a* value, representing the redness of the fillets, gradually decreased with the extension of storage time, with the fastest decrease in a* value at 35 °C, followed by a slower decreases at 25 °C, and 4 °C. In contrast, the b* values of air-fried samples at different storage temperatures displayed an increasing trend.

Measuring the pH of the meat is a crucial way to determine changes in color, flavor, odor, tenderness, and overall eating quality [27]. The changes in pH values in air-fried hairtail fillets stored at different temperatures are shown in Figure 1D. As shown in Figure 1D, the pH value of freshly air-fried samples was 6.77, while the pH value of air-fried samples at a storage temperature of 4 °C showed an increasing trend as storage progressed. pH alterations often serve as a chemical indicator of microbial spoilage in meat and related products [28]. The rise in pH during storage might be attributed to the accumulation of basic nitrogen-containing substances such as ammonia and biogenic amines, resulting from microbial or endogenous enzymatic metabolism [29,30]. The pH of air-fried hairtail fillets showed more intense fluctuations at storage temperatures of 25 and 35 °C. These variations in pH may be due to differences in the degree of offsetting of multiple reactions involving sugar, fat oxidative decomposition, and protein decomposition at various temperatures [31].

The TBARS assay quantifies the concentration of secondary lipid oxidation byproducts, with MDA serving as a representative marker [32]. In Figure 1E, the study revealed a consistent increase in MDA content across all samples with prolonged storage compared with freshly air-fried samples (with an initial MDA content of 0.64 mg MDA/kg). This increase is associated with the accumulation of hydrogen peroxide degradation products. At 4 °C, the MDA content in the samples increased slowly with increased storage time, while the samples stored at both 25 °C and 35 °C peaked at 1.29 and 1.42 mg MDA/kg on the 9th and 4th day, respectively. Throughout the entire storage period, the TBARS values determined for the three sample groups did not go over the threshold of 2 mg MDA/kg [33]. Typically, its upward tendency is associated with the accumulation of secondary oxidation products as the degree of oxidation intensifies. In addition, prior research has established that MDA commonly arises as a product of lipid oxidation due to endogenous lipid hydrolysis, free radical activity generated by specific microorganisms, and oxidative enzymes [34,35]. Therefore, the increase in TBARS values in air-fried hairtail fillets during storage might be associated with bacterial proliferation. These findings suggest that lowering storage temperatures can effectively slow down lipid oxidation in air-fried hairtail fillets.

The TVCs of air-fried hairtail fillets during storage are depicted in Figure 1F. The initial TVC of the raw sample was 2.48 log CFU/g, significantly higher than that of freshly air-fried samples (*p* < 0.05), indicating that high-temperature treatments can substantially reduce microorganisms. The TVC in all samples exhibited a noticeable rise as the storage duration increased, with a faster growth rate observed at higher temperatures. After 12 days of storage at 4 °C, the TVC exceeded the microbiological limit set for cooked meat products by the China National Food Safety Standard (GB/2726, 2016), which is 5 log CFU/g, reaching 5.35 log CFU/g. Conversely, air-fried samples stored at 25 °C and 35 °C surpassed detection thresholds on the 3rd and 6th day, respectively. These results emphasize the substantial effect of storage temperature on microbial growth in air-fried samples during storage. However, Adrah et al. [7] indicated that during the storage period, no microorganisms were detected in deep-fried samples, highlighting the importance of exploring microorganism composition in air-fried production.

In addition to the aforementioned changes, evaluating the overall acceptability of air-fried hairtail fillets is crucial for understanding consumer preferences. Figure 1G illustrates the alterations in composite sensory scores during storage. With increased storage time, different temperatures exhibited varying effects on the appearance and taste of air-fried hairtail fillets. Freshly air-fried samples had a sensory score of 9.78, signifying the best color, tender flesh, and distinctive fried flavor, garnering the highest sensory score. However, as storage time increased, the sensory score declined, attributed to a slight darkening of the color of the samples, deformation due to physical compression during vacuum packaging, and a loss of the unique aroma of air-fried hairtail fillets.

### 3.2. Microbial Analysis

#### 3.2.1. Sequencing Data Analysis

The 16S rRNA gene amplicon sequencing method is a powerful tool for characterizing microbial communities, providing comprehensive insights into their diversity and richness. To ensure accuracy, an averaged OTU table was generated using the lowest sample read number. Table 1 illustrates the α-diversity and richness outcomes for 16S rRNA amplicons from the air-fried samples. Notably, the Good’s coverage value, as presented in Table 1, exceeded 0.99, affirming the reliability of the sequencing outcomes in representing the microbial composition within the sample.

**Figure 2:**
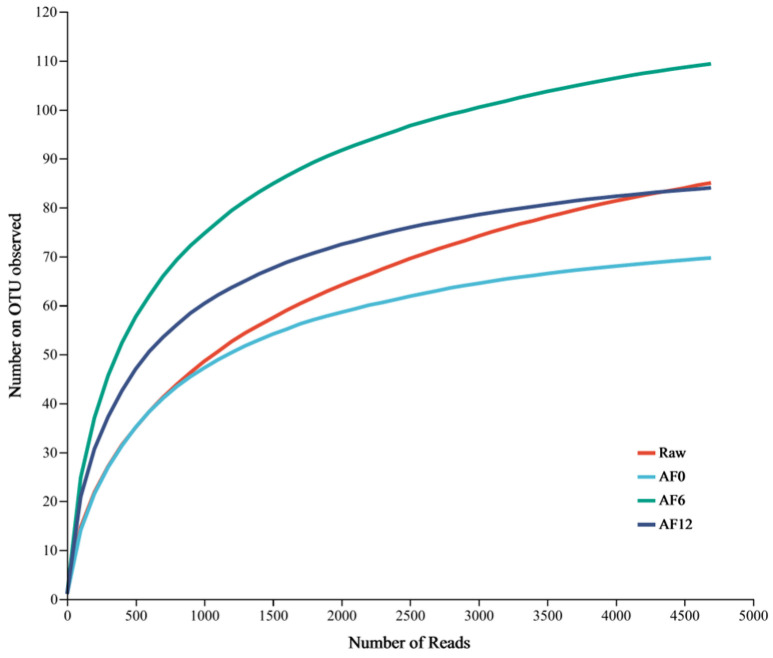
Rarefaction curves of OTUs for bacteria in air-fried samples during storage at 4 °C. Raw: raw sample; AF: air-fried sample; and 0/6/12: storage time (day).

In the initial raw samples, the species richness indices estimated by ACE and Chao1 estimators demonstrated substantial diversity with values reaching 106.25 and 105.67, respectively. However, following air frying, a significant reduction in bacterial diversity was observed, indicated by decreased ACE and Chao1 richness estimators for hairtail samples. While the ACE and Chao1 estimators of air-fried samples initially increased on day 6, they decreased by day 12 during storage at 4 °C. Higher Shannon values denote greater community diversity [36]. However, the Simpson index showed the opposite tendency. The data in Table 1 reveal decreased community diversity after air frying. Nevertheless, the community diversity of air-fried samples initially increased on day 6, followed by a decrease by day 12 during storage at 4 °C. Examination of the dilution curves indicated a consistent pattern of change in community diversity (Shannon and Simpson) and community richness (ACE and Chao1) at 4 °C, aligning with the number of microbial species. As shown in Figure 2, with increasing sequence numbers, the rarefaction curves of all samples progressively leveled off, demonstrating that the OTUs effectively represented the entire microbial population.

#### 3.2.2. Bacterial Community Composition

**Figure 3:**
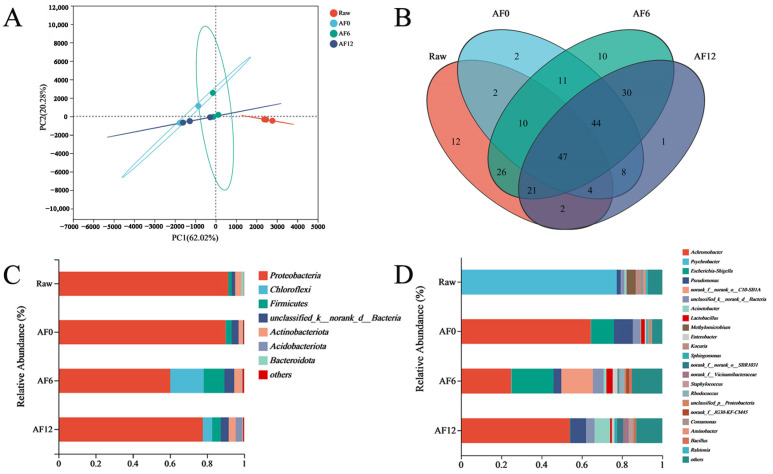
Principal component analysis (PCA) of the bacterial community of air-fried hairtail samples (**A**); Venn diagrams for numbers of shared and unique genera under different air-fried hairtail fillets during storage (**B**); relative abundance of spoilage microbiota during air-fried samples storage at 4 °C; phylum level (**C**); and (**D**) genus level. (Raw: raw sample; AF: air-fried sample; and 0/6/12: storage time (day)).

The bacterial community composition was analyzed using principal component analysis. According to Figure 3A, the findings are considerably reliable, with discernible discrepancies between PC1 (62.02%) and PC2 (20.28%) at the genus level, signifying significant distinctions. There were notable differences in the bacterial community composition between raw and air-fried samples, indicating a substantial effect of the air-frying heat method on bacterial diversity. The air-fried treatment samples tended to cluster, possibly suggesting the dominance of a specific bacterium over others as the primary spoilage bacterium, resulting in a decline in bacterial diversity.

To examine the similarities and differences among the microbiota, a Venn diagram was generated (Figure 3B), revealing 47 core OTU groups common across all samples. This highlights their potential importance during cold storage. In raw samples, 12 unique OTUs were detected in addition to the common OTUs. However, after the air-frying process, the number of unique OTUs decreased to only two. However, after 6 days of storage, the unique value soared to 10.

To comprehensively understand the microbial community dynamics during the cold storage of air-fried hairtail fillets, the investigation extended to both phylum and genus taxonomic levels. The distribution of air-fried samples at the phylum level under different storage periods is presented in Figure 3C. Key phyla included Proteobacteria, Chloroflexi, Firmicutes, Actinobacteriota, Acidobacteriota, and Bacteroidota. The dominant phyla observed were Proteobacteria, Chloroflexi, Firmicutes, and Actinobacteriota. Similarly, Zhang et al. [37] reported that Proteobacteria and Firmicutes were the dominant phyla in processed poultry during storage. Proteobacteria remained the predominant phylum during the storage period. The significant number of proteobacteria and firmicutes and their variation throughout storage suggest their importance to the sensory development of air-fried hairtail fillets. Meanwhile, after air frying, the relative abundance of Bacteroidota in hairtail samples dropped to lower than 0.1% and almost disappeared at the end of storage, a phenomenon contrasting with previous studies highlighting Bacteriodota as one of the most prevalent bacteria in seafood [38].

Figure 3D illustrates the composition and relative abundance of microbiota at the genus level. In raw samples, Psychrobacter, Methylomicrobium, and Kocuria were the predominant species, accounting for 76.99%, 4.42%, and 2.97%, respectively. However, after air frying, Achromobacter replaced Psychrobacter as the key microbial genus, constituting 64.44% of the air-fried samples, followed by Escherichia-Shigella (11.35%) and Pseudomonas (9.37%). These were three main genera in the air-fried samples. The analysis of the results suggests that the initial bacterial communities in hairtail fillets underwent substantial alterations as a direct consequence of the air-frying treatment. The composition of predominant microorganisms during storage in air-fried hairtail fillets was different. After the 12th day of storage, Achromobacter (53.98%), Pseudomonas (7.82%), and Acinetobacter (7.70%) were the major genus found in air-fried samples.

As storage time extended, the trend of variations in the relative abundance of Achromobacter and Pseudomonas was consistent with that for Proteobacteria, which decreased first and then increased. Meanwhile, the relative abundances of other bacterial genera, such as Lactobacillus and Enterobacter, increased on day 6, followed by a decline on day 12. Similar to other microorganisms, the majority of Enterobacteriaceae undergo heat sterilization during production. However, a small proportion of Enterobacteriaceae may still sustain sublethal damage after cooking before swiftly recovering, which could pose a threat to food safety [37,39]. Interestingly, Enterobacteriaceae produces aldehydes and ketones, which improve the flavor of spoiled meat [40]. The relative abundances of Acinetobacter in air-fried samples showed an increasing trend; the previous study had suggested that Acinetobacter has been described as spoilage organisms of meat products during cold periods [41]. Furthermore, it is noteworthy that Ralstonia, a recognized spoilage strain in meat products as reported by Liang et al. [42], was conspicuously absent in the air-fried hairtail fillets.

**Figure 4:**
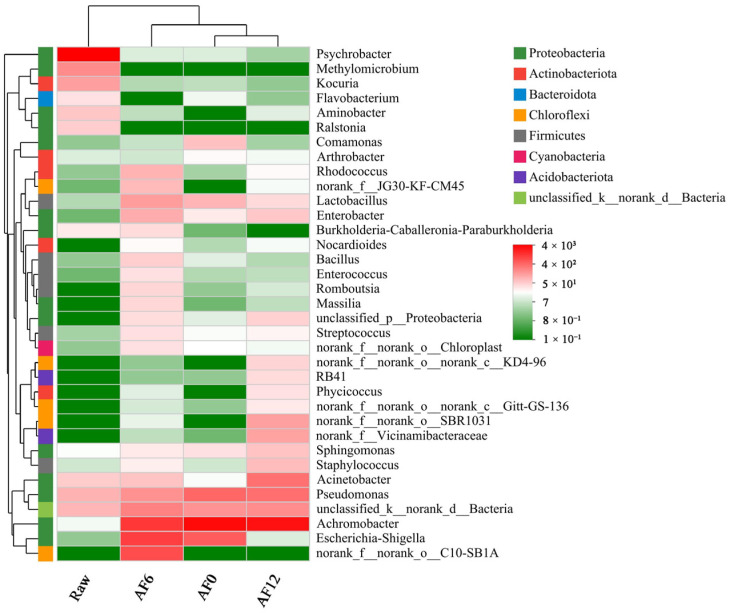
Microbial community heat map of the top 30 abundant genera during the storage period. Red squares indicate higher concentrations of the substances, while green squares showcase lower concentrations. (Raw: raw sample; AF: air-fried sample; and 0/6/12: storage time (days)).

A heat map of the top 35 genus-level phylotypes was created to display variations in bacterial dynamics and composition during cold storage. The color intensity changing from green to red indicated that as storage time passed, the relative abundance increased. As can be seen from Figure 4, the bacterial community structure of air-fried hairtail fillets changed with the increase in storage time. The proportions of genera in the raw samples, including Psychrobacter, Acinetobacter, Methylomicrobium, Kocuria, Aminobacter, and Ralstonia, considerably decreased after air frying. This result confirms the idea that the thermotolerance of these species is relatively low. Significant differences also exist in the microbial community structures of air-fried samples during the storage period. Due to its minimal requirements for complex nutrients, the genus Bacillus is widespread. In our study, the relative abundance of Bacillus on day 6 was significantly higher than that on day 0. In a previous study by Ayari et al. [43], cooked meat was observed to be a favorable environment for the growth of bacteria, particularly Bacillus cereus. During cold storage, the five most abundant genera were Achromobacter, Escherichia-Shigella, Pseudomonas, norank-d-Bacteria, and Lactobacillus on day 0. By day 6, it switched to norank-o-C10-SB1A. The proportions of Acinetobacter and norank-o-SBR1031 subsequently displayed a sudden rise on day 12.

#### 3.2.3. Comparison of Microbial Differences of Air-Fried Samples

**Figure 5:**
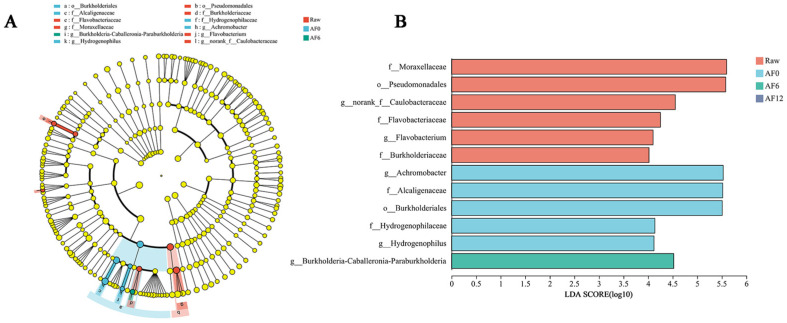
Species difference analysis. LEfSe comparison (**A**) and LDA scores (higher than 4) (**B**) on genus level in five samples. (Raw: raw sample; AF: air-fried sample; and 0/6/12: storage time (day)).

To further elucidate the dynamics of the microbial community in air-fried hairtail fillets during cold storage [44], we employed Linear discriminant analysis Effect Size (LEfSe) analysis. This occurred after defining the structural makeup of the microbiota in air-fried samples. LEfSe results indicated that there were 12 significantly different microorganisms in the air-fried samples that underwent cold storage (Figure 5A). Beyond the AF12 group, the other three groups of samples exhibited a notable prevalence of type-specific microorganisms, highlighting distinct microbial compositions associated with different processing and storage conditions. In the raw samples, our analysis revealed a significant enrichment of Moraxellaceae and Pseudomonadales. This could be attributed to the fact that Pseudomonas is a naturally-occurring spoilage bacteria frequently found in aquatic products [45]. After air frying, the significantly enriched bacteria mainly belonged to Achromobacter, Alcaligenaceae, and Burkholderiales. Alcaligenaceae, which belongs to the order Burkholderia, is well known as a lignin-derived aromatic degrader in the environment [46]. Wang et al. [47] reported that the primary order in Dezhou-braised chicken after high-temperature treatment is also Burkholderiales. Meanwhile, the only significantly enriched bacterium in the AF6 group is Burkholderia-Caballeronia-Paraburkholderia, which belongs to Proteobacteria. Previous studies [48,49] have indicated the substantial prevalence of Burkholderia-Caballeronia-Paraburkholderia in the gastrointestinal tract of triatomines and the endosphere of rice leaves, suggesting its potential role as a biological probiotic. In the Linear Discriminant Analysis (LDA) (Figure 5B), the LDA values of 12 strains from the air-fried samples exhibited values above 4.0. Notably, the LDA values for the four strains in the RAW and AF0 groups were close to 5.5, whereas the maximum LDA value observed in the AF6 group was approximately 4.5. However, no significantly enriched bacteria were detected in the AF12 group.

### 3.3. Correlation Analysis between Microbial Diversity and Environmental Factors

**Figure 6:**
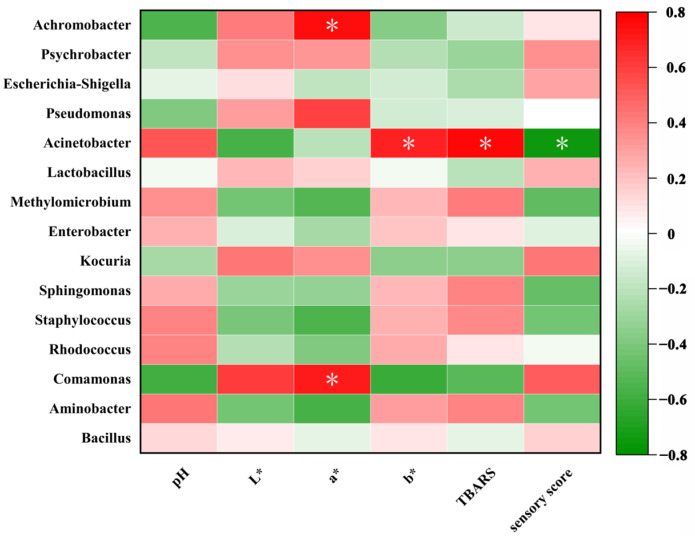
Pearson correlation analysis of microorganisms at genus levels and quality properties. The relative abundance of the genera is depicted by color intensity from green (lowest relative abundance) to red (the highest relative abundance). (Raw: raw sample; AF: air-fried sample; and 0/6/12: storage time (days)). Asterisks stand for significant differences: * *p* < 0.05.

To determine the initial causal relationships between the key characteristics and key microorganisms in air-fried hairtail fillets, we evaluated the Pearson correlation coefficients between these characteristics and bacteria (Figure 6). It was established that stronger correlations were associated more strongly with colors: A positive correlation was indicated by red, and a negative correlation by green. The results showed that Achromobacter and Psychrobacter were inversely correlated with the pH, b* value, and TBARS. They were also positively correlated with the L* value, a* value, and sensory score. Our previous results [50] proved that, in addition to being able to survive in the gastrointestinal environment, Psychrobacter has the potential to inhibit the growth of prevalent aquatic pathogens such as *S. aureus*. Meanwhile, Methylomicrobium, Enterobacter, Sphingomonas, Staphylococcus, and Aminobacter were positively correlated with pH, b* value, and TBARS, while being negatively correlated with the L* value, a* value, and sensory score. Staphylococcus, a prevalent bacterium in various ecological niches, plays a noteworthy role in meat product preservation. A noteworthy characteristic of this entity is its capacity to inhibit lipid oxidation and degrade the peroxides produced as a result of lactic acid metabolism [50]. This dual function is of significant importance in the context of meat products. In addition, a notable finding of our study was the significant (*p* < 0.05) negative correlation observed between Acinetobacter and sensory scores. This correlation underscores the pivotal role of Acinetobacter in the sensory degradation of air-fried hairtail fillets. Conversely, no significant connections were identified between the relative abundance of Bacillus and physicochemical changes, indicating a weaker association between Bacillus and the spoilage process of air-fried hairtail fillets.

## 4. Conclusions

This study investigated the physicochemical properties of air-fried hairtail fillets stored at different temperatures. It also explored the diversity of microbial communities during refrigerated storage. By analyzing these attributes and the dynamics of microbial communities, we established a correlation between physicochemical quality and critical microbial populations. This clarification of the interrelationship sheds light on the connections between physicochemical quality and microbial communities.

Throughout the storage period, we observed an increase in the pH, TVC, and TBARS, while the sensory scores declined in all samples. A reduction in storage temperature was observed to decelerate the progression of quality changes in air-fried hairtail fillets. The recommended optimum storage temperature was prescribed to be 4 °C. The predominant microbial phyla identified included Proteobacteria, Chloroflexi, and Firmicutes, with *Psychrobacter* and *Pseudomonas* being the dominant genera in raw samples. In contrast, *Achromobacter*, *Escherichia-Shigella*, and *Pseudomonas* emerged as the dominant genera throughout the entire storage duration.

This study significantly expands our understanding of microbiological spoilage and the physicochemical quality of air-fried hairtail fillets during refrigerated storage. Moreover, these findings could provide valuable insights for developing improved storage methods for air-fried fish products.

## Figures and Tables

**Table 1:** The α diversity index of bacteria in the samples. Raw: raw sample; AF: air-fried sample; and 0/6/12: storage time (days).

Sample	OTUs	Shannon	Simpson	Chao1	ACE	Good’s Coverage
Raw	85	1.79	0.30	105.67	106.25	0.99
AF0	69	1.42	0.48	78.22	76.34	0.99
AF6	109	2.40	0.26	125.68	123.80	0.99
AF12	84	2.09	0.34	90.56	89.68	0.99

## Data Availability

The original contributions presented in the study are included in the article, further inquiries can be directed to the corresponding author.
